# The Influence of Chitosan Derivatives in Combination with *Bacillus subtilis* Bacteria on the Development of Systemic Resistance in Potato Plants with Viral Infection and Drought

**DOI:** 10.3390/plants13162210

**Published:** 2024-08-09

**Authors:** Liubov Yarullina, Joanna Kalatskaja, Vyacheslav Tsvetkov, Guzel Burkhanova, Ninel Yalouskaya, Katerina Rybinskaya, Evgenia Zaikina, Ekaterina Cherepanova, Kseniya Hileuskaya, Viktoryia Nikalaichuk

**Affiliations:** 1 Institute of Biochemistry and Genetics, pr. Oktyabrya, 71, 450054 Ufa, Russia; guzel_mur@mail.ru (G.B.); evisheva@yandex.ru (E.Z.); k_cherepanova@mail.ru (E.C.); 2 Institute of Experimental Botany Named after V.F. Kuprevich, ul. Akademicheskaya, 27, 220072 Minsk, Belarus; kalatskayaj@mail.ru (J.K.); yalouskaya92@mail.ru (N.Y.); kate.rybinskaya@gmail.com (K.R.); 3 Department of Biochemistry and Biotechnology, Ufa University of Science and Technology, ul. Zaki Validi, 32, 450076 Ufa, Russia; 89173545627@ya.ru; 4 Institute of Chemistry of New Materials, The National Academy of Sciences of Belarus, 220141 Minsk, Belarus; k_hilevskay@mail.ru (K.H.); vica10bcn@gmail.com (V.N.)

**Keywords:** *Solanum tuberosum* L., *Bacillus subtilis*, potato virus, conjugates, chitosan, caffeic acid, drought, gene expression, PR proteins, proteome, induced resistance

## Abstract

Viral diseases of potatoes are among the main problems causing deterioration in the quality of tubers and loss of yield. The growth and development of potato plants largely depend on soil moisture. Prevention strategies require comprehensive protection against pathogens and abiotic stresses, including modeling the beneficial microbiome of agroecosystems combining microorganisms and immunostimulants. Chitosan and its derivatives have great potential for use in agricultural engineering due to their ability to induce plant immune responses. The effect of chitosan conjugate with caffeic acid (ChCA) in combination with *Bacillus subtilis* 47 on the transcriptional activity of PR protein genes and changes in the proteome of potato plants during potato virus Y (PVY) infection and drought was studied. The mechanisms of increasing the resistance of potato plants to PVY and lack of moisture are associated with the activation of transcription of genes encoding PR proteins: the main protective protein (PR-1), chitinase (PR-3), thaumatin-like protein (PR-5), protease inhibitor (PR-6), peroxidase (PR-9), and ribonuclease (PR-10), as well as qualitative and quantitative changes in the plant proteome. The revealed activation of the expression of marker genes of systemic acquired resistance and induced systemic resistance under the influence of combined treatment with *B. subtilis* and chitosan conjugate indicate that, in potato plants, the formation of resistance to viral infection in drought conditions proceeds synergistically. By two-dimensional electrophoresis of *S. tuberosum* leaf proteins followed by MALDI-TOF analysis, 10 proteins were identified, the content and composition of which differed depending on the experiment variant. In infected plants treated with ChCA, the synthesis of proteinaceous RNase P 1 and oxygen-evolving enhancer protein 2 was enhanced in conditions of normal humidity, and 20 kDa chaperonin and TMV resistance protein N-like was enhanced in conditions of lack of moisture. The virus coat proteins were detected, which intensively accumulated in the leaves of plants infected with potato Y-virus. ChCA treatment reduced the content of these proteins in the leaves, and in plants treated with ChCA in combination with *Bacillus subtilis*, viral proteins were not detected at all, both in conditions of normal humidity and lack of moisture, which suggests the promising use of chitosan derivatives in combination with *B. subtilis* bacteria in the regulation of plant resistance.

## 1. Introduction

Viral diseases of potato are among the main problems causing deterioration of tuber quality and loss of crop productivity [1,2]. The growth and development of potato plants and the yield of tubers depend largely on soil moisture. *Bacillus* can lead to advantageous effects on plant fitness through a broad spectrum of mechanisms, including phytohormones synthesizing and augmenting the availability of minerals to plants [3]. Some of them bring certain benefits, acting as plant-growth-promoting microorganisms (PGPM) [3,4,5].

*Bacillus* species are among the most studied biocontrol agents that contribute to the suppression of plant pathogens by antagonism and/or competition. Inhibition of pathogen growth by *Bacillus* spp. involves mechanisms such as competition for nutrients and space, production of antibiotics, hydrolytic enzymes, siderophores, and/or induction of systemic resistance [6,7,8]. Bacterial bioagents can be used for protecting various plant species against viral diseases [9]. The disadvantages of biopesticides include a relatively low rate of eradication of pathogens and high sensitivity to adverse environmental factors [10]. In our opinion, it is very important to increase the stability and diversity of the spectrum of activities of microbiological preparations for plant protection from different biotic and abiotic environmental factors by supplementing the bacterial strain with biologically active substances [4,5,11].

Considering the limitations and uncertainties associated with the use of microbial strains, alternative approaches should be introduced which could improve plant health directly by stimulating plant growth, or indirectly by helping beneficial microbes. For instance, use of the compounds thuricin 17 and lipo-chitooligosaccharides has shown promising effects on crop production even under extreme environmental growth conditions; these compounds are suggested to be suitable alternatives to chemical pesticides that could sustainably promote crop growth by successfully circumventing some of the critical environmental limitations that hinder microbial efficiency [6]. Chitosan can also act as a signaling molecule for communication between PGPR and plant roots, and occasionally initiate defense against viral infection [12]. Chitosan antiviral activity in plants has been investigated previously [13,14,15]. It has been shown that chitosan can be used for promotion of the biological activity of microbiological preparations based on strains of the genus *Bacillus* [12,16,17].

Thus, *B. velezensis* RC218 in combination with chitosan has demonstrated the ability to reduce Fusarium Head Blight disease severity and accumulation of the Fusarium-derived toxin deoxynivalenol in *T. aestivum* plants in greenhouse and field trials [18]. In addition, the combination of 0.5% chitin oligomers in combination with *B. subtilis* HS93 or *B. licheniformis* LS674 significantly (by 62% and 70%, respectively) reduced the development of Phytophthora and Rhizoctonia pepper root rot compared to control plants [19].

Defense reaction against pathogens involves an increase in the production of reactive oxygen species (ROS). H_2_O_2_ can be considered as the most important molecule involved in the transmission of intracellular signals that regulate gene expression and the activity of defense systems [20]. Activation of transcription of PR-genes, encoding peroxidase (PR-9), endo-1,3(4)-beta-glucanase (PR-2), PR-4, and PR-5 (thaumatin-like protein) is an important mechanism of the biocontrol ability of *B. subtilis* MBI600 [21]. The amino acid proline takes part in the stabilization of plant membranes, the structure of proteins, and plays a role in ROS detoxification [22]. A significant accumulation of proline in plants inoculated with endophytic bacteria *B. subtilis* has been shown, and its level positively correlated with plant disease resistance [23].

However, there are also difficulties associated with the inclusion of chitosan in bacterial culture media, as it can inhibit the growth of bacterial culture due to its antimicrobial activity, as well as the dependence of the efficacy of biological products on various environmental factors, including fertility and physicochemical properties of the soil, weather conditions, etc. [18].

One of the ways to overcome the negative consequences of adding chitosan to bacterial preparations might be its modification. Phenolic compounds are involved in plant defense against herbivores, fungi, and viruses. Plants respond to pathogen attack by accumulating phytoalexins, such as hydroxycoumarins and hydroxycinnamate conjugates. The synthesis, release, and accumulation of phenolics, in particular salicylic acid, are central points of many defense strategies against pathogenic invaders [24]. It has been shown that the conjugation of chitosan and caffeic acid (ChCA) can produce materials with improved properties: antioxidant, antimicrobial, and growth-stimulating, etc. [25]. A significant growth-stimulating effect of treatment of cucumber and barley seeds with conjugates based on chitosan and hydroxycinnamic acids has been observed [26,27]. The combined treatment with *B. subtilis* and conjugates of chitin with hydroxycinnamates prime protective responses to late blight pathogen in potato plants [28].

However, the role of ChCA conjugates in the suppression of viral infection spreading remains unknown. In addition, there is no information about the ability of combinations of ChCA conjugates and bacterial agents to stimulate plant resistance to viral diseases.

In this regard, the aim of our work was to investigate the effect of chitosan conjugates with caffeic acids and their combined preparations with *B. subtilis* 47 bacteria on the gene expression of pathogen-induced proteins, proline synthesis, and changes in the proteome of potato plants during potato virus Y (PVY) infection and drought.

## 2. Materials and Methods

### 2.1. Plant, Microbe, and Virus

Micropropagated virus-free *Solanum tuberosum* L. plants cv. Breeze obtained from the Research and practical center of the National Academy of Sciences of Belarus for potato, fruit and vegetable (Republic of Belarus) were used for investigations. The potato plant micropropagation was carried out on MS (Murashige–Skoog) agar media. The adaptation of the in vitro produced plantlets to ex vitro conditions was carried out in plastic containers (4:l volume), three per variant with nine plants for each container, on peat growing media (brand name Universalny, Republic of Belarus) with nutrients in the form of mineral salts (N—350; P—410; K—470 mg/g dry weight of growing media). Plants were grown in an insect-proof greenhouse at a constant temperature of 20–21 °C, illumination of 12,000 lux and a photoperiod of 16/8 h (day/night).

The Gram-positive aerobic *Bacillus subtilis* strain 47 (BIM B-859D, brand name Karphil, Institute of Microbiology, National Academy of Sciences, Minsk, Belarus) from the Belarusian collection of non-pathogenic microorganisms was used.

Potato plants adapted within 3 weeks were treated by spraying the leaf surface with 20 mL per container: of (1) water (control plants); (2) solutions of conjugates of chitosan with caffeic acid (ChCA, 0.025 mg/mL); (3) a mixture of ChCA solutions with suspensions of *B. subtilis* strain 47 (compositions for treatment were 0.025 mg/mL ChCA + 10^7^ cells/mL of bacterial strains).

The isolate of PVYO was obtained from naturally infected potatoes in the Research and practical center of the National academy of sciences of Belarus for potato, fruit and vegetable growing in 2023. The virus was maintained by propagating potato plants with tubers in an insect-proof greenhouse at an ambient temperature of 20–21 °C, illumination of 12,000 lux and photoperiod of 16/8 h (day/night). Potato material was tested for PVY strain specification using qRT-PCR analysis.

For viral isolate preparation the PVYO-infected plant potato leaves were collected and grinded with K,Na-phosphate buffer pH 7.8. Artificial infection with PVY was carried out by mechanical inoculation with donor plant sap (200 µL of sap per leaf) 3 days after treatment with mixtures.

### 2.2. Obtaining Conjugates of Chitosan with Hydroxycinnamic Acids

The conjugates of chitosan with caffeic acid (CA) were obtained by the carbodiimide method according to the procedure described in [29]. For the synthesis of conjugates, 30 kDa oligomers of chitosan (degree of deacetylation 98.3%, degree of polymerization ~ 186) manufactured by Glentham Life Sciences (Corsham, UK), CA (M = 180.16 g/mol, Sigma-Aldrich, Burlington, VT, USA) and 1-ethyl-3-(3-dimethylaminopropyl) carbodiimide hydrochloride (EDC, Sigma-Aldrich) were used. EDC was taken in a threefold molar excess to CA. The content of CA in the synthesized conjugates was determined spectrophotometrically (Specord-50, Analytic Jena, Jena, Germany). The absorption spectrum of the conjugate was recorded in the range of 200–400 nm and the content of CA was calculated using a preliminarily built calibration graph. The degree of attachment of caffeic acid to chitosan was 5.0 ± 0.6% or 53.8 ± 7.2 μg/mg of chitosan.

### 2.3. Detection of PVY by ELISA

The presence of viral particles in plant tissues was assessed using ELISA kits produced by the Federal Potato Research Center named after A. G. Lorch (Moscow, Russia). Fresh leaf samples (0.2 g) were crushed in a mortar; the sample and conjugate buffer were in a ratio of 1:10 (weight/volume). Diluted antibodies (1:500) specific for rabbit IgG were adsorbed onto the surface of wells of polystyrene multi-well plates and incubated overnight at 4–8 °C. After incubation, excess antibodies were washed out with 200 µL washing buffer per well 3 times. Then, 100 µL of homogenized plant sample extracts were added to wells, covered with a lid and incubated overnight at 4–8 °C. After incubation, unbound material was washed away, and 100 µL of diluted conjugate (1:500) was added to each well; the plate was covered with a lid and incubated for 1 h at 37 °C. After washing of the unbound conjugate, the enzymatic reaction was initiated by adding 100 µL of a freshly prepared pNPP (para-nitrophenylphosphate) substrate solution (1 mg/5 mL) to each well. The plate was incubated at room temperature for 40–50 min, after which the reaction was stopped by adding 50 µL of 3 M H_2_SO_4_ to each well. The results of the ELISA were evaluated using a Benchmark Microplate Reader (BioRad, Berkeley, CA, USA) at λ = 490 nm.

### 2.4. Proline Content

Potato leaves (200 mg) were immersed in 2.0 mL of 3% sulfosalicylic acid and centrifuged for 20 min at 12,000× *g*. Glacial acetic acid and ninhydrin-based reagent were added to an aliquot of the supernatant in a 1:1:1 ratio and heated for 60 min at 90 °C on a heat shaker with constant stirring (300 rpm). The optical density was measured on spectrophotometer (Jasko, Tokyo, Japan) at a wavelength of 515 nm [30,31].

### 2.5. Water Deficiency Application

The modeling of soil water deficiency conditions was started 3 days after artificial infection of leaves with PVY and continued for 3 months until the appearance of mini-tubers. The moisture content of the peat growing media (40–45% soil water capacity) was achieved by reducing plant watering. In the control, plants were grown at an optimum humidity of 80–85% of the soil water capacity. The regulation of optimum soil humidity and water deficiency conditions was carried out by the weight method, as well as using a moisture meter TR 001 (Renke, Jinan, China). Leaf samples—2 leaves from the 3rd and 4th level of the stem above the infected leaf of each plant variant—were collected 14 days after infection when symptoms of leaf damage appeared, mixed, and used for measurement.

### 2.6. Quantitative Real-Time PCR (qPCR) to Investigate Virus Accumulation

RNA isolation from liquid nitrogen-fixed potato leaves was performed using the reagent kit “PhytoSorb” (Synthol, Moscow, Russia) according to the method recommended by the developers [https://www.syntol.ru/catalog/dlya-vyyavleniya-fitopatogenov/, accessed on 1 July 2024].

The qRT-PCR was performed using specific primers for PVX CP (Coat Protein) and PVY PIPO (Pretty Interesting Potyviridae open reading frames (ORF)) genes by using the kit “Potato Virus X and Potato Virus Y” (Synthol, Moscow, Russia), according to the manufacturer’s protocols on a CFX Connect real-time PCR Detection System device (BioRad Laboratories, Hercules, CA, USA). Three independent biological replicates were performed for each experiment.

### 2.7. RNA Extraction and Real-Time PCR

Total RNA was isolated from plants with Lira^®^ reagent (Biolabmix, Moscow, Russia) according to manufacturer’s protocol on the 3rd day post infection. The synthesizing of first-strand cDNA and the real-time procedure were performed as described previously [31]. The expression of genes was shown as a fold change normalized to the transcription of the reference gene StAct encoding potato actin. iCycler iQ5 Real-Time Detection System 181 equipment and IQ5 optical system software, version 2.1 (BioRad, Berkeley, CA, USA) were used. The primers used for qPCR are shown in Table 1. The efficiency of primers was assessed using 10-fold cDNA dilution series.

### 2.8. Measuring of Protein Content

The protein content in the samples was determined by the Bradford method, using bovine serum albumin as a standard [32]. Absorption was measured at 595 nm.

### 2.9. Two-Dimensional Electrophoresis

The leaf homogenate was resuspended in a buffer solution (0.7 M sucrose, 0.5 HEPES-KOH (pH 7.5), 0.1 M KCl, 2% mercaptoethanol, 1 mM EGTA, 1 mM PMSF, 0.1 mM sodium orthovanadate), incubated for 30 min at 4 °C. Proteins were extracted with a phenol solution. Phenol saturated with Tris-HCl (2 mL) was added to 1 mL of a protein solution in acetone; the resulting mixture was incubated at −20 °C for 30 min, then centrifuged for 30 min at 200× *g*. Proteins from the phenolic phase were precipitated with a fourfold volume of 0.1 M ammonium acetate in ethanol at −20 °C for 10 h.

The resulting precipitate was washed three times with ammonium acetate and dissolved in a lysis buffer (8M urea, 2M thiourea, 1% CHAPS, 30 mM DTT, 20 mM Tris, 0.3% ampholyte solution).

Isoelectric focusing of proteins was performed on a Protean IEF system (BioRad, USA). To separate proteins by their isoelectric point, we used ready-made 7 cm strips (BioRad, USA), pH range 3–10. Before focusing, passive rehydration was performed for 12 h at 20 °C. Focusing was carried out at a voltage of 4000 V (20,000 V h) for 22 h, then the voltage was maintained at 500 V until the end of the process. After isoelectric focusing, the strips were kept for 15 min successively in solutions of 2% dithiothreitol and 2.5% iodoacetamide in buffer solutions with 25% glycerol, then washed in 0.025 M Tris-glycine buffer, pH 8.3.

SDS-electrophoresis was performed in 10% PAGE. Strip and marker proteins on filter paper were placed on a polyacrylamide gel and spilled in 1% agarose on Tris-glycine buffer solution. Electrophoresis was carried out at a voltage of 90–120 V, the gels were stabilized in 50% ethanol for 10 min, then stained with 0.1% Coomassie G-250 solution.

### 2.10. Mass Spectrometry

Protein identification was performed on a MALDI Bruker Ultraflex II mass spectrometer (Germany). Tryptic hydrolysis of the protein in the gel was carried out for 18 h at 37 °C, stopping with the addition of 7 µL of 0.7% trifluoroacetic acid solution. For mass spectrometric analysis, 0.5 µL of a hydrolyzed sample solution and 0.5 µL of 10 mg/mL dihydroxybenzoic acid in 50% acetonitrile and 0.7% trifluoroacetic acid were mixed directly on the target of the mass spectrometer. The mass spectra were recorded in reflex mode by summing the signals received at 1000 laser pulses.

If necessary and possible, the fragmentation spectra of individual peptides (MS/MS) were recorded using tandem LIFT mass spectrometry. The accuracy of measuring monoisotope masses is no worse than 70 ppm, the accuracy of measuring fragment masses is no worse than 1.5 Da. Protein identification was performed using the MASCOT service in the SWISS-PROT protein sequence database and the local MASCOT service using data related to the studied taxa deposited in the GenBank NCBI data bank.

### 2.11. Statistical Processing

At least 5 biological groups in 3 technical repetitions were tested in biochemical trials, and at least 15 in tests of gene expression. Data presented are mean values with standard errors (±SE). In order to assess the statistical significance of the differences among some biological groups, Duncan’s test was performed. The level of significance used for data analysis is 95%.

## 3. Results

### 3.1. Susceptibility of Leaves to PVY

**Figure 1:**
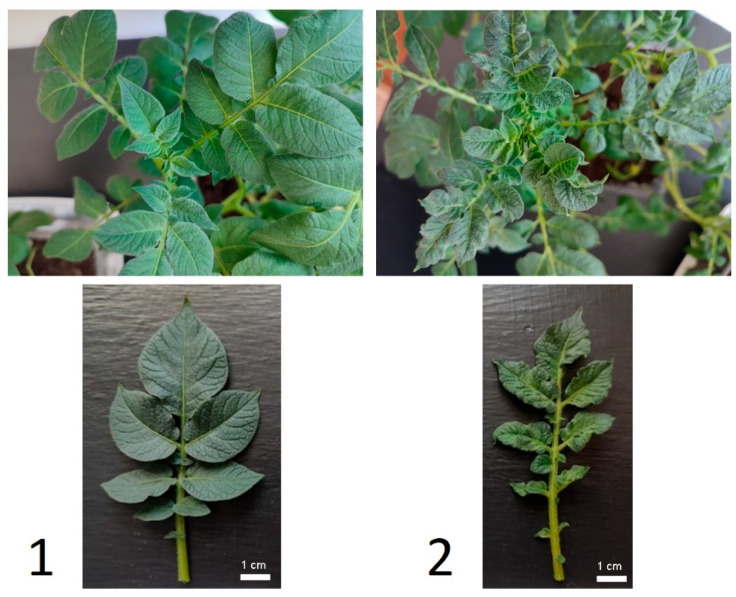
The appearance of a leaf of a healthy (1) and PVY-infected (2) potato plant.

Ten days after infection, potato plants showed wrinkled mosaics, chlorosis and necrotic spots on infected leaves (Figure 1). RT-PCR analysis confirmed the presence of PVY in potato leaves of previously infected experimental variants.

**Figure 2:**
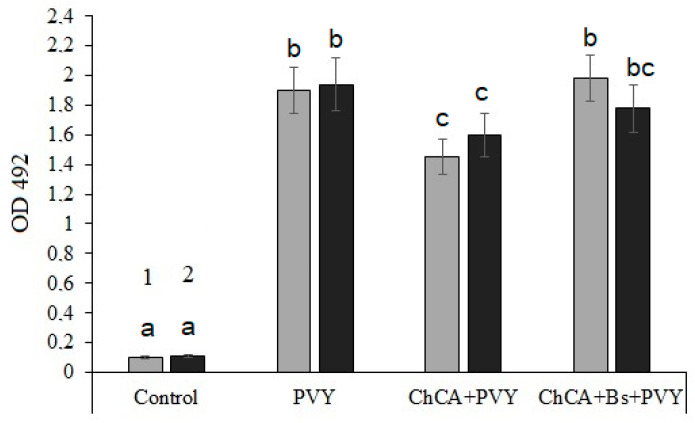
Detection of PVY by ELISA in the sap of potato leaves after treatment with ChCA and *B. subtilis* 47 in normal conditions (1) and water deficiency (2). Different letters denote significantly different values.

The ELISA results demonstrated that the treatment of plants with ChCA reduced the degree of infection of potato leaves with PVY both in conditions of optimal and insufficient soil moisture (by 23.7% and 16.3%, respectively). Combinations of *B. subtilis* + ChCA did not affect the degree of infection of PVY plants under optimal humidification conditions, but in conditions of moisture deficiency the combination of *B. subtilis* + ChCA significantly reduced the level of infection (Figure 2).

### 3.2. Content of Proline and Proline Synthase Activity

**Figure 3:**
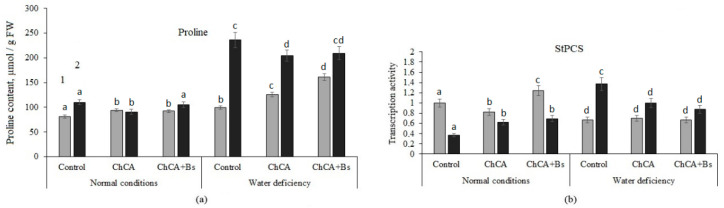
The influence of ChCA and *B. subtilis* 47 on the proline content (**a**) and pyrroline-5-carboxylate synthase transcription level (**b**) in healthy (1) and PVY-infected (2) potato plants on the 10th day after PVY inoculation. Different letters denote significantly different values.

Application of ChCA alone and *B. subtilis* 47 with ChCA increased proline content in relation to untreated plants under optimal conditions by 25.4% and 62.6%, respectively under soil water deficiency. Moreover, the mixture of *B. subtilis* 47 with ChCA contributed to the higher accumulation of proline as compared with ChCA alone. Plant infection with PVY led to an increase in proline content, but when ChCA was used, the amount remained at the level of uninfected ChCA-treated plants. Soil water deficiency in combination with PVY infection increased proline accumulation 1.4 times. Meanwhile, both variants of treatments allowed reduction in the proline content in PVY-infected plants by 13.8% and 11.5%, respectively, compared to non-treated infected plants. There were no significant differences between conjugate ChCA alone or mixture with *B. subtilis* 47 in healthy plants under optimal moisture conditions and in infected plants under water deficiency (Figure 3).

Treatment with ChCA and ChCA + *B. subtilis* stimulated the accumulation of proline in healthy plants under conditions of moisture deficiency. In treated plants, when infected with PVY against the background of soil moisture deficiency, the proline content increased by two times (Figure 3).

The accumulation of proline correlated with an increase in the transcriptional activity of the pyrroline-5-carboxylate synthase (P5CS) gene (Figure 3b). An increase in proline content under stress is associated with the activation of its synthesis catalyzed by pyrroline-5-carboxylate synthase (P5CS) and pyrroline-5-carboxylate reductase (P5CR) [19].

### 3.3. Transcriptional Activity of PR-Genes

The influence of *Bacillus* and conjugates of chitin with caffeic acid on the level of transcripts of genes *StPR1*, *StPR3*, *StPR5*, *StPR6*, *StPR9*, *StPR10* and *StMT* encoding the protein *PR*-1 (marker of salicylate-dependent pathway), *StPR3* (chitinase), *PR*-5 (thaumatin-like inhibitor), *PR*-6 (proteinase inhibitor, marker of jasmonate-dependent pathway), *PR*-9 (peroxidase), *PR*-10 (ribonuclease), and *StMT* (methyltransferase), respectively (Table 1) in intact and PVY-infected plants was investigated.

**Figure 4:**
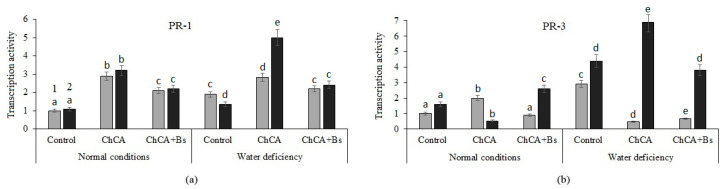
The effect of the ChCA and *B. subtilis* 47 on the relative number of transcripts of the PR-1 ((**a**), main protective protein) and PR-3 ((**b**), chitinase) genes in healthy (1) and PVY-infected (2) plants under normal conditions and under conditions of water deficiency. Different letters denote significantly different values.

Treatments of ChCA and ChCA + *B. subtilis* plants stimulated the accumulation of *StPR1* gene transcripts in healthy and infected plants with optimal soil moisture (Figure 4a). In conditions of water deficiency, the transcriptional activity of the StPR1 gene decreased in untreated infected plants. At the same time, in infected plants treated with ChCA and ChCA + *B. subtilis*, the level of accumulation of transcripts of the *StPR1* gene in drought conditions was significantly higher than the control (Figure 4a).

Under optimal humidification conditions and under conditions of water deficiency, an increase in the transcriptional activity of the *StPR3* gene was observed in plants infected with PVY (Figure 4b). The multidirectional effect of ChCA plant treatment on the accumulation of *StPR3* gene transcripts in YVK-infected plants was revealed: with optimal moisture supply it decreased, and with a lack of moisture it was stimulated. When treated with a mixture of ChCA + *B. subtilis*, the level of transcriptional activity of the *StPR3* gene in infected plants increased both under optimal conditions and under conditions of water deficiency (Figure 4b).

**Figure 5:**
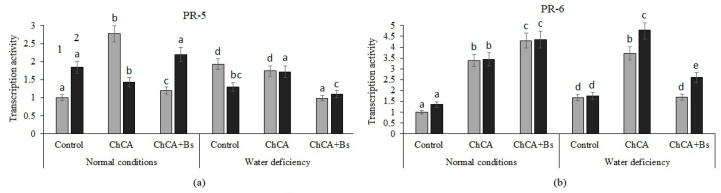
The effect of the ChCA and *B. subtilis* 47 on the relative number of transcripts of the PR-5 ((**a**), thaumatin-like protein) and PR-6 ((**b**), protease inhibitor) genes in healthy (1) and PVY-infected (2) plants under normal conditions and under conditions of water deficiency. Different letters denote significantly different values.

ChCA treatment led to a significant increase in the transcriptional activity of the PR-5 gene in healthy plants, and—in combination with *B. subtilis*—in infected potato plants, under optimal humidification conditions (Figure 5a). Treatment with ChCA and a mixture of ChCA + *B. subtilis* increased the transcriptional activity of the PR-6 gene several times in healthy and infected plants under normal conditions, whereas in conditions of water deficiency, a similar result was revealed only when treating with ChCA (Figure 5b).

**Figure 6:**
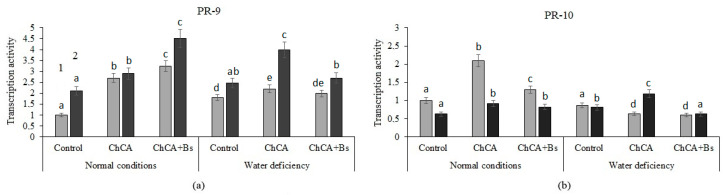
The effect of the ChCA and *B. subtilis* 47 on the relative number of transcripts of the PR-9 ((**a**), peroxidase) and PR-10 ((**b**), ribonuclease) genes in healthy (1) and PVY-infected (2) plants under normal conditions and under conditions of water deficiency. Different letters denote significantly different values.

Treatment with ChCA alone and a mixture of ChCA + *B. subtilis* enhanced the accumulation of PR-9 gene transcripts in healthy and infected plants under optimal humidification conditions and under conditions of water deficiency (Figure 6a).

The transcriptional activity of the PR-10 gene was enhanced when treated with ChCA conjugate in healthy potato plants under optimal conditions and in infected plants under conditions of water deficiency (Figure 6b). The stimulating effect of co-treatment with ChCA conjugates with *B. subtilis* bacteria on the transcriptional activity of PR-10 genes was detected in healthy and infected plants under optimal conditions.

**Figure 7:**
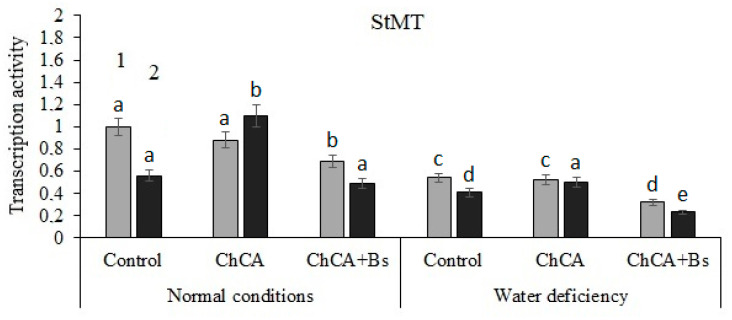
The effect of the ChCA and *B. subtilis* 47 on the relative number of transcripts of the StMT (methyltransferase) gene in healthy (1) and PVY-infected (2) plants under normal conditions and under conditions of water deficiency. Different letters denote significantly different values.

The stimulating effect of ChCA conjugate treatment on the transcriptional activity of the *StMT* gene in infected plants under normal conditions and under conditions of water deficiency was revealed (Figure 7).

### 3.4. Proteome

One of the approaches to detecting changes in gene expression in plants under the influence of biologics treatment and infection with pathogens can be the study of the proteome of plant tissues. By two-dimensional electrophoresis of *S. tuberosum* leaf proteins followed by MALDI-TOF analysis, 10 proteins were identified, the presence of which in the leaves differed depending on the variant of the experiment (Table 2).

As can be seen from Table 2, treatment with ChCA, ChCA + *B. subtilis* 47, infection, and drought changed the spectrum of leaf proteins compared to control.

## 4. Discussion

### 4.1. Susceptibility of Leaves to PVY

The ChCA treatment of plants reduced the degree of infection of potato leaves under both optimal and insufficient soil moisture conditions. The combination of *B. subtilis* bacteria with ChCA conjugate significantly reduced the level of plant infection in conditions of moisture deficiency (Figure 2).

It is known that colonization by beneficial microorganisms causes a physiological state of the host plant called priming. The “primed” status of the plant allows for stronger and faster protective reactions against subsequent invasion of pathogens, which manifests itself as a common sign of systemic resistance induced by beneficial microorganisms [33].

Chitin, chitosan, and their oligomers are active elicitors of plant immunity [34]. Oxycinnamic acids, under the influence of stress factors of various natures, can be included in the phenylpropanoid pathway, changing the direction of synthesis of their own derivatives [35], thus enhancing the formation of phenolic compounds involved in the mechanisms of increasing plant resistance [36]. It can be assumed that the combined treatment of chitosan conjugates with caffeic acid and *B. subtilis* bacteria contributes to the formation of earlier and more intense protective reactions upon contact with the pathogen.

We have found that the treatment of ChCA plants reduced the PVY content in potato leaves both under conditions of optimal moisture and water deficiency (Figure 2). Treatment with *B. subtilis* + ChCA contributed to a decrease in the titer of viruses in plant tissues only under conditions of water deficiency. At the same time, the anti-stress effect of ChCA and *B. subtilis* + ChCA on infected and drought-affected plants was revealed, as judged by a decrease in proline synthesis (Figure 3).

### 4.2. Content of Proline and Proline Synthase Activity

It is known that the regulation of proline levels is crucial for maintaining the osmotic potential of tissues, which is important in drought conditions. The additional synthesis of this amino acid increases the overall resistance of plants to abiotic stresses, leading to increased nonspecific resistance. Proline is considered as a metabolic signal regulating redox homeostasis and the expression of certain stress response genes.

In our studies, the accumulation of proline under the influence of treatment with ChCA and ChCA + *B. subtilis* (Figure 2a) during infection with PVY under normal conditions and with moisture deficiency correlated with an increase in the transcriptional activity of the pyrroline-5-carboxylate synthase (P5CS) gene (Figure 2b), which was shown earlier [36].

Proline synthesis can be induced by exogenous signaling molecules, brassinosteroids [37], salicylic acid [38], and chitosan [39]. The protective effect of proline is due to its participation in the stabilization of membranes, the structure of protein molecules, and a decrease in ROS levels. In addition, proline is involved in the regulation of many cellular processes and is one of the indicators of activation of systemic plant protection, performing a signaling function in the interaction of plants with pathogens [29].

### 4.3. Transcriptional Activity of PR-Genes

One of the mechanisms of the protective action of biologics based on *Bacillus* bacteria is the enhancement of ROS production [40] and the indirect induction of PR protein gene expression [41]. In our studies, the transcriptional activity of chitinase (PR-3), thaumatin-like protein (PR-5), protease inhibitor (PR-6), and peroxidase (PR-9) genes was increased in infected plants treated with *B. subtilis* 47 in combination with ChCA under optimal conditions. It has been shown that bacteria of the genus *Bacillus* produce chitinases and glucanases into the culture medium, hydrolyzing chitin and peptidoglycans, which are an important component of the cell wall of microorganisms, and also induce the formation of other protective compounds [42]. In response to infection by viruses, fungi, and bacteria, the accumulation of mainly chitinases of the third type (PR-3) occurs in the intercellular fluid of plant tissues. The main protective mechanism of the PR-5 protein is associated with an increase in the permeability of pathogen membranes. An increase in the expression of the protease inhibitor gene (PR-6) is a marker of the development of SAR [43].

The combined stress caused by viral infection and water deficiency led to significant expression of the genes *StPR1*, *StPR3*, *StPR6*, *StPR9*, and *StPR10* in the variant with ChCA treatment; however, when using a mixture of ChCA + *B. subtilis* 47, the transcriptional activity of PR proteins was lower.

It is known that plants peroxidases of class III belonging to the family of protective proteins of the PR-9 family are involved in strengthening cell walls due to oxidative reactions that catalyze the polymerization of phenolic compounds in the lignin of cell walls, increasing their resistance to destruction by phytopathogens. Proteins of the PR10 family can function as fungicides, and this ability, associated with nuclease activity, can be manifested both by direct action on the pathogen during penetration into the cell and destruction of cellular RNAs [44], and by participating in the hypersensitivity reaction completely [29]. Trachinia plants show that *B. subtilis* B26 causes an increase in the activity of methyltransferases involved in the maintenance and regulation of plant DNA methylation [45]. This suggests the involvement of bacteria of the genus *Bacillus* in the regulation of plant resistance at the epigenetic level. In general, the plant’s resistance to a particular stress factor is determined by the expression of many genes encoding protective proteins.

### 4.4. Proteome

Chloroplast 20 kDa chaperonin, belonging to the group of HSP-like proteins, was found in all plants. Chloroplast chaperones are a structurally and functionally diverse and flexible system capable of adapting to specific substrates under various external conditions through regulation at both translational and posttranslational levels [46]. One of the mechanisms of such adaptation, characteristic of chloroplasts, is the formation of chaperonin hetero-oligomers [47]. This makes chaperonins capable of working not only with plant proteins, but also with proteins of plant symbionts or pathogens. There is also evidence of the association of chaperonins with an increase in the activity of superoxide dismutase (SOD) [https://www.uniprot.org/uniprot/Q8LK52, accessed on 1 July 2024].

Numerous literature data [48] indicate the exceptional importance of chaperonins for the development of the infectious process. In our experiments, there was a significant decrease in the chaperonin content in the leaves relative to the control under the influence of lack of moisture. Moreover, the content of this protein decreased when infected with the Y-virus, especially strongly in combination with ChCA alone and *B. subtilis* + ChCA. Apparently, one of the mechanisms of plant resistance is the regulation of protein synthesis, and this mechanism is involved in both biotic and abiotic stress.

In animals and plants, innate immunity is regulated by nucleotide-binding proteins, including proteins with a leucine-rich repeat (NB-LRR), which are involved in pathogen recognition and activation of protective reactions of the host cell [49]. One of these proteins is TMV resistance protein N-like. As shown in tobacco plants, the tobacco N gene is part of the plant resistance class (R) genes associated with the Toll-interleukin-1 receptor/nucleotide-binding site/leucine-rich repeat (TIR-NBS-LRR), which provide resistance to tobacco mosaic virus (TMV) [50]. It encodes two transcripts, N(S) and N(L), formed by alternative splicing, which are necessary for the formation of plant resistance to TMV. N-gene expression is mediated by the 126 kDa C-terminal helicase domain of the TMV replicase protein [51].

In conditions of infection with the virus, we noted a decrease in the content of this protein in the leaves, which may be one of the manifestations of the infectious process. Moreover, the content of this protein decreases when treated with various resistance inducers or lack of moisture, showing its involvement in various systemic ways of implementing the plant’s response to stress.

Dehydroascorbate reductase (DHAR), belonging to the glutathione-S-transferase (GST) superfamily, is a key enzyme involved in ascorbate metabolism and catalyzes the glutathione-dependent reduction in oxidized ascorbate (dehydroascorbate, DHA) [52]. This enzyme participates in the reaction of plants to oxidative stress [53]. The ascorbate–glutathione pathway is considered to be key in the metabolism of H_2_O_2_ [54]. It has been shown that overexpression of dehydroascorbate reductase stimulates the resistance of tobacco plants to oxidative stress [55] and rice plants to root nematodes [56]. DHA treatment of plants causes an early and stable transcriptional response, including the induction of genes associated with plant stress responses, immunity, redox metabolism, and synthesis of secondary metabolites. DHA is able to activate induced systemic plant resistance mediated by ROS production and activation of the salicylate pathway [56]. In our experiment, in all treatment options, except for ChCA, there was a significant decrease in the DHA content relative to the control.

With regard to 50S ribosomal protein L4, it is known that many ribosomal proteins are involved in various cellular processes unrelated to ribosomes and protein synthesis [57]. Thus, *E. coli* RPL4 allosterically regulates RNase E-dependent degradation of RNA by inhibiting its cleavage and stabilizing mRNA. This ribosomal protein participates in the stress response, increasing the decay time of PR gene transcripts [57]. In addition, it has been shown to inhibit the transcription and translation of the S10 operon of mRNA and participation in rRNA processing. In our experiment, in all treatment options with inducers of resistance, lack of moisture, and viral infection, there was a significant decrease in the content of RPL4 relative to the control.

Proteinaceous RNase P 1 has enzymatic activity that catalyzes the 5′ maturation of tRNA precursors, and is also capable of cleaving tRNA-like structures [58]. Most plant viruses are RNA viruses, many of which contain a functional tRNA-like structure. Rpp30 provides resistance to a wide range of fungal and bacterial pathogens by increasing the expression of protective genes and the production of reactive oxygen species. RNase P functions either as a catalytic ribonucleoprotein (RNP) or as an RNA-free polypeptide that catalyzes RNA processing, primarily the maturation of 5′-tRNA [59]. In plants, the activity of RNase P is provided exclusively by PRORP proteins [60]. *Arabidopsis thaliana* PRORP2 has been shown to interact with TRM1A and B methyltransferases. Studies of PRORP proteins have allowed the development of ribonuclease, which serves as an effective means of protecting plants from viruses [61]. It has also been shown that the production of extracellular ribonuclease increases the resistance of cucumber plants to TMV [62].

In our experiments, the content of RNase P was elevated relative to control in plants infected with potato virus Y and plants pretreated with ChCA, as well as in plants grown under conditions of lack of moisture. Other treatments and their combinations led to a multidirectional change in the content of this enzyme. This indicates the complexity and versatility of the processes in which RNase P is involved. It is known that many plant RNases are multifunctional, and the direct relationship between their ribonucleolytic activity and antiviral protection still needs to be clarified [62].

2-methylenefuran-3-one reductase is also a protein involved in many protective processes, activated by treatment with resistance inducers and infection with pathogens [63]. Its participation in the implementation of resistance to *P. palmivora* and *Taphrina deformans* is shown. The synthesis of this protein is induced during maturation and inhibited by auxin. This protein participates in the biosynthesis of furaneol, a volatile compound, which can participate in the transmission of protective signals. The homolog of this protein is NADPH-dependent oxidoreductase, which promotes the detoxification of stromal reactive carbonyls formed during oxidative stress [64]. This protein can use NADH and NADPH as electron donors to catalyze redox reactions [65].

In our experiments, the content of this protein significantly decreased relative to the control when infected with the Y-virus in the absence of treatment with resistance inducers. In conditions of lack of moisture, on the contrary, there was an increase in its synthesis. The combination of lack of moisture with various treatments and virus infection caused a multidirectional change in the content of this protein, depending on the variant of the experiment.

In a number of experimental variants, there was a change in the content of oxygen-evolving enhancer protein (OEEP), a chloroplast protein involved in the generation of ROS. At normal humidity, its content decreased in the ChCA and *B. subtilis* + ChCA treatment options, and with a lack of moisture in all treatment options with the exception of ChCA.

OEE1 and OEE2 are important members of a complex that participates in the photooxidation of water during the light reactions of photosynthesis. It is known that an oxidative burst is a signal for a hypersensitivity reaction, which is closely related to the response of plants to contact with a pathogen. It is believed that the complex of enhancer proteins exhibits high sensitivity to stress [66]. There is evidence of the relationship of the oxidative explosion protein RPH1 with the resistance of arabidopsis plants to *Phytophthora brassicae* and potatoes to *Phytophthora infestans* [67]. There are numerous phosphorylation sites in the OEEP protein sequence [68], which suggests its participation in the regulation of the activity of various enzymes.

Another protein, the content of which varied significantly and in different directions depending on the variant of the experiment, was stem-loop-binding protein. Replication-dependent histone mRNAs end in a highly conserved structure consisting of a 26-nucleotide stem loop. Stem-loop-binding protein (SLBP) is an evolutionarily conservative protein with no known homologs, which interacts with the stem-loop both in the nucleus and in the cytoplasm and mediates nuclear cytoplasmic transport, as well as 3′–terminal processing of snRNP U7 pre-mRNA [69]. SLBP is also regulated during the cell cycle, accumulating when cells enter the S-phase and rapidly degrading when cells exit the S-phase [70]. It has been shown that impaired SLBP function leads to the inability of cells to transition from proliferation to differentiation [71]. Currently, there is no information in the literature on the involvement of SLBP plants in the stress response and the formation of resistance. The multidirectional change in its content during various treatments in our experiments does not allow us to draw an unambiguous conclusion about its mechanism of action in the formation of plant resistance; this should be the subject of further study.

The synthesis of 70 kDa heat shock-related protein is regulated by plant hormones traditionally associated with the stress response. Abscisic acid, jasmonates, ethylene, and salicylic acid have a negative effect on the expression of HSP70. Cytokinins, on the contrary, activate most HSP70s. In addition, HSP70 genes are subject to increased regulation in response to many viruses. Thus, an increase in HSP70 levels has been reported in response to tobacco mosaic virus (*N. benthamiana*; cytosolic HSP70 genes), turnip mosaic virus (*Brassica rapa*; two cytosolic HSP70 genes) and pea seed mosaic virus (*Pisum sativum*; two cytosolic HSP70 genes). Induction of HSP70 may represent a host response to the synthesis of a large number of exogenous proteins, and some resistance genes are reported to encode regulators of HSP70 expression. However, according to newer data, the expression of HSP70 in the plant is caused directly by pathogens. An indirect confirmation of this is the absence of a change in the expression of HSP70 when infected with the vine leaf coagulation virus, which has its own HSP70 gene [72].

Intercellular transport of plant viruses is mediated by specific virus-encoded factors called displacement proteins and viral envelope proteins that can form complexes with HSP70. It is believed that these complexes mediate the interaction of the virus with the cytoskeleton and facilitate its transfer into and through plasmodesma. There are reports of a positive effect of HSP70s on replication of Chinese wheat mosaic furovirus and cow fungus heavy mosaic virus. In addition, *Nicotiana tabacum* plants exposed to heat shock and inoculated with potato Y-virus showed higher HSP70 gene expression and higher virus content than the corresponding control plants inoculated at standard temperature [72].

As the results of our experiments showed, in plants infected with potato Y-virus, the content of HSP70 remained at the level of control plants. When treated with resistance inducers in the absence or in the presence of viral infection, as well as with a lack of moisture, the content of this protein decreased. The data obtained are consistent with the ideas about the important role of HSP70 in the development of viral infection, and also show the possibility of effective regulation of HSP70 levels by inducers of plant resistance.

It can be noted that in conditions of normal humidity, intensive synthesis of proteinaceous RNase P 1 is observed in plants treated with ChCA and infected with PVY. In this treatment variant, the plants had the highest resistance to virus infection. Probably, the RNase activity prevented the development of the virus and the normal course of the infectious process, causing plant resistance.

During ChCA treatment and PVY infection, higher oxygen-evolving enhancer protein 2 content was also observed, almost reaching the control level, than in other treatment options under normal humidity conditions. This protein is associated with an oxidative burst. This indicates the key role of the hypersensitivity reaction in the development of resistance of potato plants to PVY in conditions of normal humidity.

In conditions of lack of moisture, the highest resistance of plants to the virus was also observed during ChCA treatment. With this treatment, the content of 20 kDa chaperonin increased most strongly in drought conditions. This protein belongs to the group of HSP-like proteins, so its activation in response to water deficiency is likely. Moreover, having chaperone activity, this protein can contribute to the assembly of viral particles. Thus, the effect of enhancing the synthesis of 20 kDa chaperonin when infected with a virus in drought conditions may be due to either the virus or the drought, or both. However, with other treatments involving virus infection, both under normal conditions and during drought, there was no pronounced increase in the content of this protein. Coupled with the fact that the greatest resistance to the virus is observed during ChCA treatment in drought conditions, we can conclude that the HSP-like functional activity of chaperonin is one of the key factors contributing to the increased resistance of ChCA-treated plants to the virus in drought conditions.

In addition to chaperonin, ChCA treatment in drought conditions (which most effectively increased plant resistance to the virus) increased the content of TMV resistance protein N-like approximately twofold relative to control in both infected and uninfected plants. Thus, if RNase appears to be the main “antiviral” protein activated by ChCA in conditions of normal humidity, then in conditions of lack of moisture it appears to be the TMV resistance protein N-like that may suppress the development of PVY.

Finally, the proteins—the content of which varied depending on the variant of the experiment—turned out to be virus envelope proteins or their precursors (coat protein, polyprotein). These proteins were intensively accumulated in the leaves of plants infected with potato virus Y. Treatment with ChCA reduced the content of these proteins in the leaves, and treatment with *B. subtilis* + ChCA led to a much more significant decrease in their content. Interestingly, in conditions of lack of moisture, the accumulation of virus proteins in infected leaves was much less pronounced. Both in conditions of normal humidity and with a lack of moisture, the strongest decrease in the content of viral proteins was observed in plants pretreated with *B. subtilis* + ChCA.

## 5. Conclusions

Thus, the treatment of plants with chitosan conjugate with caffeic acid (ChCA) and its mixture with the *B. subtilis* contributed to an increase in the resistance of potato plants to viral infection both in normal conditions and with a lack of moisture. ChCA treatment reduced the content of PVY coat proteins in the leaves. In plants treated with ChCA together with *B. subtilis*, viral proteins were not detected at all. The protective effect of the treatments was associated with the activation of transcription of genes encoding PR proteins: the main protective protein (PR-1), chitinase (PR-3), thaumatin-like protein (PR-5), protease inhibitor (PR-6), peroxidase (PR-9), and ribonuclease (PR-10), as well as qualitative and quantitative changes in the plant proteome. At the proteome level, plant resistance is mediated by proteinaceous RNase P1 and oxygen-evolving enhancer protein 2 in conditions of normal humidity, and 20 kDa chaperonin and TMV resistance protein N-like in conditions of lack of moisture. The revealed activation of the expression of marker genes of systemic acquired resistance PR-1 and induced systemic resistance PR-6 under the influence of joint treatment with *B. subtilis* and ChCA indicates the synergistical development of protective reactions. The data obtained suggest the prospects of using chitosan conjugate in combination with *B. subtilis* bacteria to increase plant resistance to viral diseases with a lack of soil moisture.

## Figures and Tables

**Table 1:** Primers used for PCR.

Product	Gene	NCBI Number	Direct Primer	Reverse Primer
Actin	StAct	X55749	gat-ggt-gtc-agc-cac-ac	att-cca-gca-gct-tcc-att-cc
PR-1	StPR1	AY050221	tgg-gtg-gtg-gtt-cat-ttc-ttg-t	cat-tta-att-cct-tac-aca-tca-taa-g
Chitinase, PR-3	StPR3	U49970	ttc-tgg-atg-aca-gca-cag-gat	ggc-gtc-cat-tgc-cca-at
Thaumatin-like protein, PR-5	StPR5	AY737317	ccc-gtt-tga-cat-tga-cct-ttg	cga-ata-cgg-tgg-aac-atg-ga
Proteinase inhibitor, PR-6	StPR6	JX683427	ggg-aaa-gaa-tat-gct-caa-gtt-at	aat-tct-cca-tca-tct-tcc-act-g
Peroxidase, PR-9	StPR9	M21334	gta-atc-ctg-ccg-cac-aac-t	gca-gca-aaa-tct-cca-agg-aa
Ribonuclease, PR-10	StPR10	AF500589	ctc-gct-aac-cct-tct-gtc-tat-g	caa-cac-gtc-ctg-atc-atc-tct-c
Methyl transferase	StMT	XM_006356514	ggc-aat-gga-cat-taa-ccg	tca-aga-aga-ggc-aaa-gca-g
Proline carboxylate synthase	StPCS	XM015308529	tta-aag-agg-acg-gag-ctt-gc	cag-tgc-atc-agg-tcg-tga-ct

**Table 2:** The presence of some proteins in potato leaves. C—control, ChCA—chitosan-caffeic acid conjugate, PVY—potato virus Y, Bs—*B. subtilis*. “-”—protein was not detected.

Protein Name in Uniprot	pI	MW	Protein Content, μg/g Fresh Weight											
			Normal Conditions						Soil Moisture Deficiency					
			C	ChCA	Bs ChCA	PVY	PVY ChCA	PVY Bs ChCA	C	ChCA	Bs ChCA	PVY	PVY ChCA	PVY Bs ChCA
20 kDa chaperonin	5	25	14.7	12	10.8	6.8	3.1	3.8	4.8	8.4	3.7	4.6	9.2	10
TMV resistance protein N-like	6	25	9.6	4.3	6.8	9.4	4.4	5	4.6	8.8	4.1	4.7	8.2	6
Dehydroascorbate reductase	6	25	8	10	4	5	4.2	4.2	3.3	3.7	4.5	4.3	4.6	4.2
50S ribosomal protein L4	6	30	18.3	7.4	7.4	7.4	12.4	9.4	12.4	7.6	7.1	7.1	8	15
Proteinaceous RNase P 1	6.5	35	3.3	3.1	4.9	3	7.4	-	9	4	6.2	-	3.5	4.7
2-methylene-furan-3-one reductase	6	40	11.6	11	11	3.7	12.8	13.4	18.5	19	-	-	3.8	3.1
Oxygen-evolving enhancer protein 2	5	25	9.6	4.4	3.5	5	7.2	6.8	8.2	9	-	-	-	-
Stem-loop binding protein of 41 kDa a	6	40	8.4	12.1	13.5	4.5	6.6	-	4	11.4	-	-	4	-
70 kDa heat shock-related protein	4	75	6.2	8.4	-	8.4	-	3.1	4.6	3.1	-	-	3.6	-
Coat protein, partial [Potato virus Y]/Polyprotein	7	30	-	-	-	17.7	11.8	-	-	-	-	4.1	3.2	-

## Data Availability

The original contributions presented in the study are included in the article, further inquiries can be directed to the corresponding author.
